# Depression Recognition Using Machine Learning Algorithms With Eye Tracking, Visual Evoked Potentials, and Auditory P300 Among Chinese Medical Students

**DOI:** 10.1155/da/8637398

**Published:** 2025-11-20

**Authors:** Rongxun Liu, Jinnan Yan, Shisen Qin, Peng Luo, Yuanle Chen, Luhan Yang, Guangjun Ji, Chao Wang, Xuebing Huang, Fei Wang, Yong Meng, Yange Wei

**Affiliations:** ^1^Department of Early Intervention, Mental Health and Artificial Intelligence Research Center, The Second Affiliated Hospital of Henan Medical University, Henan Mental Hospital, Xinxiang 453002, China; ^2^School of Public Health, Henan Medical University, Xinxiang 453003, China; ^3^School of Psychology, Henan Medical University, Xinxiang 453003, China; ^4^Department of Neuroelectrophysiology, The Second Affiliated Hospital of Henan Medical University, Xinxiang 453002, China; ^5^NHC Key Laboratory of Mental Health, National Clinical Research Center for Mental Disorders, Peking University Sixth Hospital, Peking University Institute of Mental Health, Beijing 100191, China; ^6^Department of Early Intervention, Nanjing Brain Hospital, Nanjing Medical University, Nanjing 210029, China; ^7^Department of Psychiatry, Yale School of Medicine, New Haven, Connecticut 06511, USA

**Keywords:** auditory P300, Chinese medical students, depression, eye-tracking, machine learning, visual evoked potential

## Abstract

**Background:**

Current assessment of depression primarily relies on psychological scales. Although the use of machine learning in depression has grown, limited reports are available on multiple neurophysiological measurements. We employed machine learning algorithms incorporating eye tracking, visual evoked potentials (VEPs), and auditory P300 to classify depression among Chinese medical students.

**Methods:**

A total of 66 students with depression and 72 matched controls were recruited; eye tracking, VEPs, and auditory P300 data were collected. Descriptive analyses and group comparisons were performed between the depression and control groups. Then, multivariate logistic regression (LR) analysis was conducted to evaluate the relationship between eye tracking, VEPs, and auditory P300 features and Patient Health Questionnaire-9 (PHQ-9) scores. Furthermore, the study employed six classifiers to differentiate between depression and nondepression. Five-fold cross-validation was employed. Model performance was assessed using receiver operating characteristic (ROC) curves, area under the curve (AUC), precision, accuracy, recall, and F1 score. We applied SHapley Additive exPlanations (SHAP) values to explain the model.

**Results:**

Depression group was characterized by lower response search scores, higher *D* values, and prolonged P100 latencies in both eyes. No significant differences were observed in auditory P300 features. Random forest (RF) classifier demonstrated superior classification performance relative to the other five machine learning algorithms. Models utilizing combined features showed enhanced performance compared with those based solely on eye tracking or VEP features. Utilizing the SHAP method, we identified that P100 latency in the right eye was the most significant feature across all machine learning models.

**Conclusions:**

Chinese medical students with depression exhibited reduced responsive search scores and extended P100 latencies, suggesting impairments in attention and visual information processing associated with depression. The combined eye tracking and VEPs proved to be more effective than single features for distinguishing depression and nondepression. P100 latency in the right eye may be the most significant predictor of depression.

## 1. Introduction

The medical students worldwide have considerably elevated rates of depression in comparison to their peers who have other academic disciplines. A comprehensive meta-analysis encompassing data from 167 cross-sectional studies and 16 longitudinal studies across 43 countries indicates that 27.2% of medical students experience depression [1]. In China, the prevalence of depression among medical students exceeds 30% [2]. They frequently encounter academic pressures, including rigorous examinations, intensive clinical training, and scientific research obligations. Additionally, they may experience relationship difficulties with partners and a lack of time for family and friends, all of which can contribute to an elevated risk of depression. Individuals suffering from depression often exhibit enduring feelings of sadness and a diminished interest in activities. They also display a negative information bias across various cognitive processes, such as attention, memory, and interpretation [3–5]. Individuals impacted by these challenges may exhibit diminished academic performance and impaired social skills, which can contribute to broader societal issues [6]. Furthermore, depression is linked to low self-esteem, suboptimal academic outcomes, strained personal relationships, diminished empathy toward patients, as well as increased risks of alcohol and substance abuse, and suicidal tendencies among medical students. Consequently, early identification is essential for the effective management and prevention of depression. While current assessment techniques for depression predominantly depend on self-report instruments and clinical observations, both of which are subject to inherent subjective limitations regarding accuracy and reliability. To address these challenges, researchers have increasingly focused on objective neurophysiological measurements to enhance the precision of depression identification.

Neurophysiological measurements such as eye tracking, visual evoked potential (VEP), and auditory P300 have garnered significant attention recently due to their noninvasiveness, convenience, and cost-effectiveness [7]. This study selected three modalities due to their ability to provide complementary insights into the various stages of information processing deficits that characterize the pathophysiology of depression, ranging from early sensory processing to higher-order cognitive and attentional control. Eye tracking, in particular, has proven to be highly effective in identifying attentional biases associated with depression. Individuals with depression tend to exhibit prolonged fixations on dysphoric images compared to neutral or positive stimuli, thereby indicating a negative attentional bias. This maladaptive attentional pattern, considered a key factor in the maintenance of the disorder, can also manifest as altered gaze patterns, such as increased saccades and fewer overall fixations during certain visual tasks [8]. Furthermore, eye tracking research has demonstrated that the inability to disengage attention from negative stimuli is predictive of diminished stress recovery in individuals with depression [9]. VEP provides an objective measure of the brain's response to basic environmental stimuli, with the latency of VEPs serving as an indicator of arousal levels in the visual cortex [10]. Specifically, VEPs offer a perspective on the integrity of early, preattentive sensory processing. Research examining the effects of different rotation angles of visual stimuli on VEPs has demonstrated that early components are directly associated with the initial stages of visual information processing [11]. The neurophysiological evidence suggests that individuals with depression exhibit altered visual perception, potentially associated with dysfunctions in visual information processing [10]. These findings indicate fundamental deficits at the initial stages of sensory registration, which may precede the cognitive biases observed in depression.

The auditory P300 component is extensively utilized to examine cognitive processes due to its high temporal resolution [12]. It is closely related to sensory-motor and cognitive behaviors generated by salient stimuli following ordinary continuous sequences [13]. As a marker of higher-order cognitive dysfunction, the P300 reflects the allocation of attentional resources and the updating of working memory. Furthermore, abnormalities in the P300 component, such as prolonged latency and reduced amplitude, are thought to reflect deficits in higher-order cognitive resource scheduling and allocation in individuals with depression, manifesting as decreased information processing efficiency, dysregulated attentional control, and impaired emotional regulation [14]. Previous research has indicated a reduction in P300 amplitude in individuals with depression compared to healthy controls [15]. Moreover, the P300 component has been found to correlate with the severity of depressive symptoms [16]. Collectively, these modalities provide a complementary and multilayered perspective on the neurocognitive mechanisms underlying depression. They encompass distinct stages of information processing, ranging from initial perceptual engagement (as measured by VEP), attentional bias (assessed through eye tracking), to higher-order cognitive evaluation and resource allocation (indexed by P300). These stages are intrinsically interconnected, for example, reduced efficiency in early processing, as evidenced by VEP latency, may impair subsequent attentional and cognitive functions, resulting in more pronounced deficits in higher-level processes as indicated by the P300. In depression research, a singular neurophysiological measurement has been investigated. Studies have identified abnormal neurophysiological features in schizophrenia, bipolar disorder, and major depressive disorder to explore cognitive impairments [17–19]. However, limited reports are available on depression among medical students in China. Consequently, this study selected eye tracking, VEP, and auditory P300 as objective neurophysiological measurements.

Moreover, recent advancements in computational methodologies, particularly in machine learning algorithms, demonstrate considerable potential in the identification of depression [20]. The ability of machine learning to analyze complex datasets and identify latent patterns offers an innovative approach that could complement and potentially enhance traditional diagnostic methods [21]. Machine learning algorithms have significantly expanded the potential of biomarkers by facilitating the integration of complex data from diverse sources. In the domain of mental health research, machine learning models, especially those utilizing supervised learning methodologies, have shown significant efficacy in differentiating individuals with depression from those without the condition [22]. These models are capable of processing extensive datasets that include clinical and biological characteristics, thereby improving the precision of depression screening [23]. While prior studies have explored the utility of eye-tracking, VEP, and auditory P300 separately for depression identification, fewer studies have investigated the integration of these features. Considering the highly heterogeneous nature of depression, utilizing multiple neurophysiological measurements—including eye tracking, VEP, and auditory P300 data—through machine learning methodologies could enhance the detection and classification of depression among Chinese medical students.

The primary objective of this study was to explore the potential of integrating eye tracking, VEP, and auditory P300 metrics with machine learning algorithms to identify depression in Chinese medical students. Specifically, this research aimed to address the following questions: (1) whether eye tracking, VEPs, and auditory P300 features demonstrate alterations in Chinese medical students with depression; (2) which features correlate with depression severity? and (3) can eye tracking, VEPs, and auditory P300 features effectively differentiate between depression and nondepression using machine learning algorithms? If so, which specific features hold relative importance for identifying depression? We hypothesize that integrating multiple neurophysiological measurements with machine learning techniques could facilitate the development of objective methods for identifying depression among Chinese medical students.

## 2. Methods

### 2.1. Study Design

**Figure 1:**
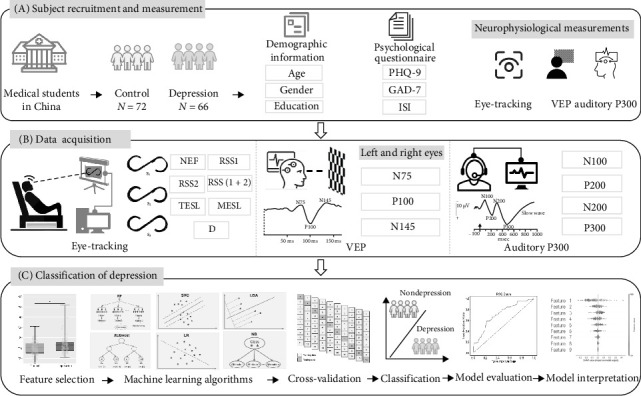
Study design. (A) A total of 138 Chinese medical students were recruited for this cross-sectional study. We collected psychological questionnaires, eye tracking, and visual evoked potential (VEP) data. (B) After data preprocessing, we extracted eye tracking, VEP, and auditory P300 features during specific task performances. (C) Eye tracking, VEP, and auditory P300 features with significant differences were selected as input features. Six machine learning models, including random forest (RF), linear discriminant analysis (LDA), naïve Bayes (NB), eXtreme gradient boosting (XGBoost), logistic regression (LR), and support vector classification (SVC), were trained to build classification models. Each algorithm was evaluated with five-fold cross-validation. Finally, we evaluated the performance of all models and assessed the importance of the selected eye tracking, VEP, and auditory P300 features.

This study recruited a cohort of 138 medical students from Henan Province, China, between March and April 2024. Initially, we conducted a statistical comparison of eye tracking, VEPs, and auditory P300 characteristics between the depression and control groups. Subsequently, we investigated the association between these neurophysiological features and depression. Finally, we employed machine learning-based classification techniques to differentiate between the depression and control groups using eye tracking, VEPs, and auditory P300 features. We conducted a comparative analysis of the classification performance between single-modality data-eye tracking, VEPs, and auditory P300 and their combined feature set. SHapley Additive exPlanations (SHAP) was used to interpret the predictions of machine learning models (see Figure 1).

### 2.2. Participant Recruitment

A cohort of 138 participants comprising 66 individuals with depression and 72 age-, gender-, and education-matched controls was recruited from Henan Medical University, Henan province, China. The inclusion criteria for the depression group were as follows: (1) participants aged between 18 and 30 years; (2) Patient Health Questionnaire (PHQ-9) score exceeding 5; and (3) the ability to complete psychological assessments, eye tracking, VEP, and auditory P300 examinations. Age-, gender-, and education-matched controls (*N* = 72) were recruited from Henan Medical University. The inclusion criteria for the control group were as follows: (1) aged between 18 and 30 years; and (2) a PHQ-9 score below 5, confirming minimal anxiety and low impact of insomnia on daily functioning. Participants were excluded if they met any of the following criteria: (1) history of cognitive impairment; (2) alcohol dependence, substance abuse, or mental disorder; and (3) history of neurological diseases or significant abnormalities in neurological examinations. The study was approved by the institutional review boards of the Second Affiliated Hospital of Xinxiang Medical University (Approval Number XYEFYLL-2023-35-4) in accordance with the Declaration of Helsinki's ethical principles of medical research involving human subjects. All enrolled participants provided written informed consent.

### 2.3. Psychological Assessment

The PHQ-9 is a brief, reliable, and validated instrument for the screening and assessment of depression severity. In this study, we assessed depression symptoms using the PHQ-9, which demonstrated good internal consistency, with a Cronbach's α coefficient of 0.89. Regarding the assessment of anxiety severity, the Generalized Anxiety Disorder-7 (GAD-7) questionnaire, a seven-item tool with a scoring range from 0 to 21, where higher scores indicate greater anxiety severity, was employed. The Cronbach's α for the GAD-7 was 0.92. The Insomnia Severity Index (ISI) is a widely used seven-item self-report questionnaire that assesses overall insomnia severity on a 5-point Likert scale, with higher scores reflecting greater symptoms of insomnia. The Cronbach's α coefficient of ISI was 0.90, indicating high internal consistency. In the current study, cutoff scores of 5 for PHQ-9, 5 for GAD-7, and 8 for ISI were adopted to detect depression, anxiety, and insomnia symptoms, respectively.

### 2.4. Neurophysiological Measurements

#### 2.4.1. Eye Tracking

On the day of physiological measurements, participants were instructed to refrain from vigorous physical activity, caffeine, or alcohol. All participants were comfortably seated in a quiet, temperature-controlled environment maintained at 22–25°C. The eye tracking was observed and recorded using the Shanghai Dikang DEM-2000 eye movement detector (Shanghai Dikang, China). All participants freely viewed static images with their left eye. The eye tracking test commenced by positioning the participant in a semireclined position on a calibrated testing chair, ensuring that their line of sight aligned with a display screen positioned 20 cm away at a 33° horizontal angle. The camera detecting corneal reflections of infrared light was employed to monitor eye movements. An LCD monitor was used to present target images for the eye tracking tasks. Precise adjustments were conducted to align the center of the participant's left eye with the center of the screen. Subsequently, a calibration procedure was implemented, guiding the participant to focus on designated points presented on the screen. This facilitated precise calibration of the eye tracking system. This calibration process was repeated until consistent and reliable tracking was attained. A nine-point calibration was conducted before the initiation of experiments. Based on the reflection of infrared light on the cornea, eye-tracking images were recorded on the videotape. Three horizontally oriented *S*-shaped images were projected directly onto the individual's eyes on a screen 25 cm away. During the initial 15 s, the *S*-shaped image 1 was presented on the screen. Subsequently, two slightly different *S*-shaped images 2 and 3 were displayed, each for a duration of 15 s. Following these presentations, participants were asked if they detected any perceived differences between the latter two *S*-shaped images 2 and 3 and the initial *S*-shaped image. Eye-tracking data were automatically captured and analyzed using a digital eye-tracking recording system.

The study measured 15 eye tracking features for the three images, including the number of eye fixations (NEFs), responsiveness of search scores (RSSs), *D* scores, mean eye scanning length (MESL), and total eye scanning length (TESL). Specifically, NEF is defined as the total number of points in the eye fixation pattern 1 within a 15 s period, where each fixation point has a duration exceeding 200 ms. RSS scores are calculated by dividing *S*-shaped images 2 or 3 into seven areas. The maximum RSS score for each image is seven points, and the total RSS score for *S*-shaped images 2-3 is 14 points. RSS variations on images 2 and 3 are represented as RSS1, RSS2, and RSS (1 + 2), respectively. The *D* scores are calculated using the discriminant analysis formula:

  $$\begin{array}{c} D=10.265-\;\left[\left(0.065\;\times \;NEF\right)+\left(0.871\;\times \;RSS\right)\right] \end{array}$$

MESL measures the average extent of eye movement, whereas TESL indicates the overall extent of the eye fixation point.

#### 2.4.2. Visual Evoked Potential

In an acoustically shielded dark room, all subjects were required to be physically relaxed and maintain stillness throughout the experiment. Each participant underwent a 10-min adaptation period to the ambient room lighting. The VEPs were recorded using the Nihon Kohden MEB-9404C evoked potential recorder (Nihon Kohden, Tokyo, Japan). In accordance with the 10–20 International System, VEP signals were recorded using gold-cup electrodes, with the active electrode positioned at Oz, the reference electrode at Fpz, and the ground electrode at Pz. The Checkerboard Reversal Pattern was employed to assess VEPs. To obtain transient responses, checkerboard patterns with 100% contrast were presented on a television monitor and underwent contrast reversal at a frequency of two reversals per second. The VEP recordings were conducted at a distance of 120 cm using the optoelectronic stimulator, Galileo Mizar Sirius, at high (15' checks) and low (55' checks) spatial frequencies. The VEP waveform exhibited a triphasic waveform, characterized by peaks approximately at 75, 100, and 145 ms following the alteration of the visual stimulus. The peak latencies of the N75, P100, and N145 components were systematically recorded. Each eye, identified as left (L) and right (R), underwent two separate tests, denoted as 1 and 2, respectively. Consequently, this procedure yielded a total of 12 distinct VEP features, specifically L1, L2, R1, and R2 for each of the N75, P100, and N145 components.

#### 2.4.3. Auditory P300

In an acoustically quiet and comfortable environment, participants were instructed to maintain physical relaxation while reclining in a chair. The auditory P300 was measured with an oddball paradigm attention task using a Dantec Keypoint 4 evoked potential instrument (Dantec Keypoint 4, Denmark). According to the International Society for Clinical Electrophysiology of Vision standards, the reference electrode was located on Cz, a second reference electrode was placed on the right earlobe (M2), and FPz was utilized as the ground electrode during recordings. The target stimulus was presented at an intensity of 90 dB and a frequency of 4000 Hz. In contrast, the nontarget stimuli were composed of pure tones at 1000 Hz with an intensity of 80 dB. Acoustic stimuli were delivered in a random sequence, with target and nontarget stimuli comprising 20% and 80%, respectively. Participants received short tone bursts binaurally and were instructed to press a button when the target was detected. The analysis time was set at 600 ms. Subsequently, we calculated the latencies and amplitudes of the N100, N200, P200, and P300 components at electrode sites Cz and Fpz, which may reflect different stages of auditory processing. In total, 16 auditory P300 features were analyzed in the study.

### 2.5. Statistical Analysis

The sample size for this study was estimated using G^⁣^*∗*^^Power 3.1.9.7 software. The specific parameters were set as follows: test family: *t* tests; statistical test: difference between two independent means (two groups); type of power analysis: a priori: compute required sample size; α error probability: 0.05; power (1-β error probability): 0.80. The Kolmogorov–Smirnov one-sample test was applied to evaluate the normality of continuous variables. Continuous data that followed a normal distribution were presented as the mean ± standard deviation (SD), whereas nonnormally distributed continuous data were reported as medians with interquartile ranges (IQRs). Categorical data were demonstrated as frequencies and percentages. The aforementioned eye tracking, VEP, and auditory P300 analyses yielded 43 features for group discrimination analyses. Independent samples *t* tests were utilized to compare continuous variables, while chi-square tests (*χ*^2^ tests) were used to assess categorical variables. For significantly altered features, multivariate logistic regression (LR) analysis was used to detect independent predictors of depression. Receiver operating characteristic (ROC) curve analyses were used to assess the predictive ability of features for identifying depression. Statistical analyses were performed using SPSS 25.0 software, with statistical significance set at a two-tailed *p*-value of <0.05.

### 2.6. Machine Learning Algorithms for Classification

We implemented six machine learning algorithms for binary classification: random forest (RF), linear discriminant analysis (LDA), naïve Bayes (NB), support vector machine (SVM), LR, and extreme gradient boosting (XGBoost). Eye tracking, VEP, and auditory P300 features with statistically significant group differences were selected as model inputs. Prior to model training, *Z*-score normalization was implemented within each fold of the cross-validation process to prevent data leakage from the test folds and to address the issue of varying data units, thereby enhancing model convergence and reducing feature bias. This normalization process standardized all features to have a mean of 0 and a SD of 1. A nested stratified five-fold cross-validation strategy was employed to optimize hyperparameters and assess model performance. The inner loop performed hyperparameter tuning using GridSearchCV, aiming to identify the optimal parameter combination that maximizes the area under the curve (AUC) ROC, while the outer loop was used to estimate the generalization performance of each model. The hyperparameter search spaces for each model were defined as follows: for RF, n_estimators = [10, 100, 200, 500, 1000] and max_depth = [None, 5]; for LDA, solver = [‘lbfgs', ‘liblinear', ‘saga'] and shrinkage = [None, ‘auto', ‘log', 0.1, 0.5, 1.0]; for NB, var_smoothing = [1e–9, 1e–8, 1e–7, 1e–6]; for SVM, C = [0.1, 1, 10, 100, 1000], kernel = [‘linear', ‘rbf', ‘poly']; for LR, C = [0.01, 0.1, 1, 10, 100], penalty = [‘l1', ‘l2', ‘elasticnet'], solver = [‘lbfgs', ‘liblinear', ‘saga']; and for XGBoost, n_estimators = [100, 200, 500, 1000] and learning_rate = [0.1, 0.05, 0.025, 0.01]. Depression was classified as binary, with a PHQ-9 score of ≥5 indicating the presence of depression (absent depression = 0, and depression = 1). The model performance evaluation metrics included AUC, ROC, accuracy, precision, recall, and F1 score. Finally, this study utilized the SHAP module to rank the contribution of each feature to the classification model. SHAP is a powerful framework rooted in cooperative game theory [24]. It assigns a unique Shapley value to each feature, precisely quantifying how much each feature contributes, both positively and negatively, to the final prediction for every individual. This allows for a granular understanding of the model's decision-making process, moving beyond simple feature rankings to reveal the direction and magnitude of each feature's influence on the classification outcome. The most significant features were identified and visualized utilizing the SHAP Python package. All machine learning procedures were implemented using the Python sklearn package version 1.2.1 (https://scikit-learn.org/).

## 3. Results

### 3.1. Participant Demographics

According to sample size calculation, at least 128 participants were necessary to achieve sufficient statistical power. To enhance statistical power and the reliability of the results, a total of 66 medical students with depression (average age 20.45 years, 50 females and 16 males) and 72 matched controls (average age 20.89 years, 56 females and 16 males) were enrolled. Data were tested for normal distribution via the Shapiro–Wilk *W*-test (*p* > 0.05). There were no significant differences in age, gender, or education level between groups (*p* > 0.05). The depression group showed higher scores on the PHQ-9, GAD-7, ISI, and PSS scales, compared with controls (*p* < 0.05). The demographic characteristics of participants are presented in Table 1.

### 3.2. Significant Changes of Eye Tracking, VEP, and Auditory P300 Features in Depression

**Figure 2:**
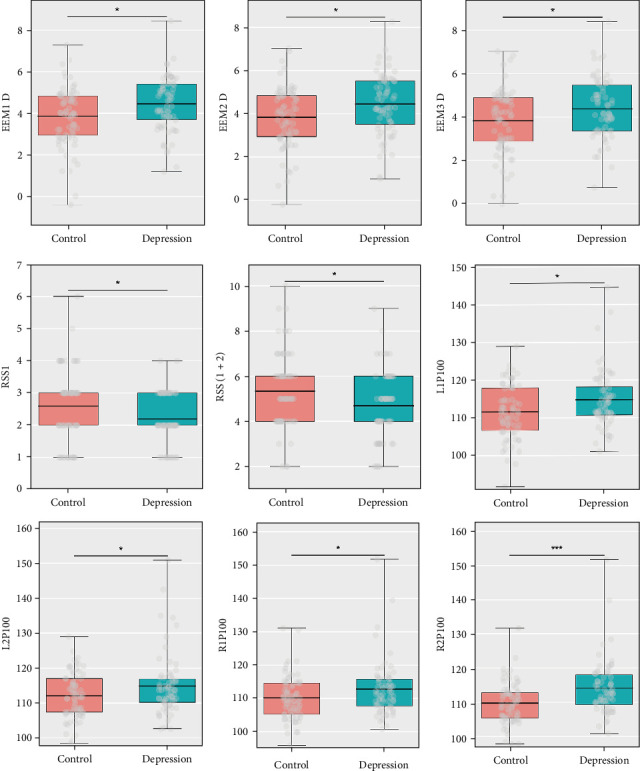
Significant differences in the eye tracking and visual evoked potential features between control and depression among Chinese medical students. *⁣*^*∗*^*p* < 0.05; *⁣*^*∗∗*^*p* < 0.01; *⁣*^*∗∗∗*^*p* < 0.001. EEM, eye movement; RSS, responsiveness of search scores; L1P100, left eye 1, P100; L2P100, left eye 2, P100; R1P100, right eye 1, P100; R2P100, right eye 2, P100.

In terms of eye tracking features, the depression group exhibited lower values in RSS1 and RSS (1 + 2), while demonstrating higher values in EEM1 D, EEM2 D, and EEM3 D, compared to the control group. For VEP features, the P100 latencies in both eyes (R1P100, R2P100, L1P100, and L2P100) were significantly prolonged in the medical students with depression. No significant differences were observed between the depression and control groups regarding the latencies or amplitudes of N100, N200, P200, and P300 at the Cz or Fpz electrode sites. For details, see Table 1 and Figure 2.

### 3.3. Multivariate LR Analysis

**Figure 3:**
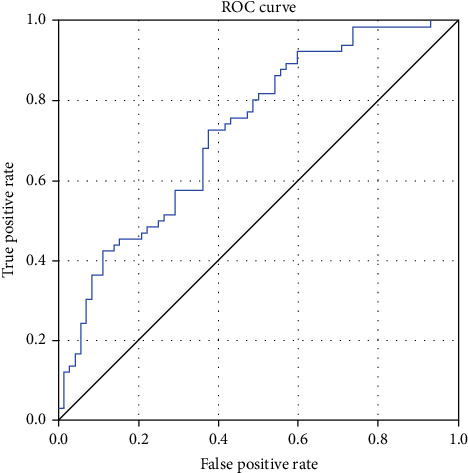
The receiver operating characteristic curve is plotted for multivariate logistic regression in the recognition of depression.

Based on the significantly altered eye tracking and VEP features, we conducted a multivariate LR analysis to identify predictors of depression. The analysis revealed that EEM1 D (95% CI: 1.001–1.741, *p* = 0.049), R1P100 (95% CI: 0.798–0.991, *p* = 0.034), and R2P100 (95% CI: 1.083–1.368, *p* = 0.034) were significant independent factors associated with depression.  Table 2 and Figure 3 show detailed information on binary LR analysis. Specifically, the combination of EEM1 D, R1P100, and R2P100 could predict depression with a sensitivity of 72.73% and a specificity of 62.50% (ROC AUC = 0.725, 95% CI: 0.641–0.808).

### 3.4. Classification Results

**Figure 4:**
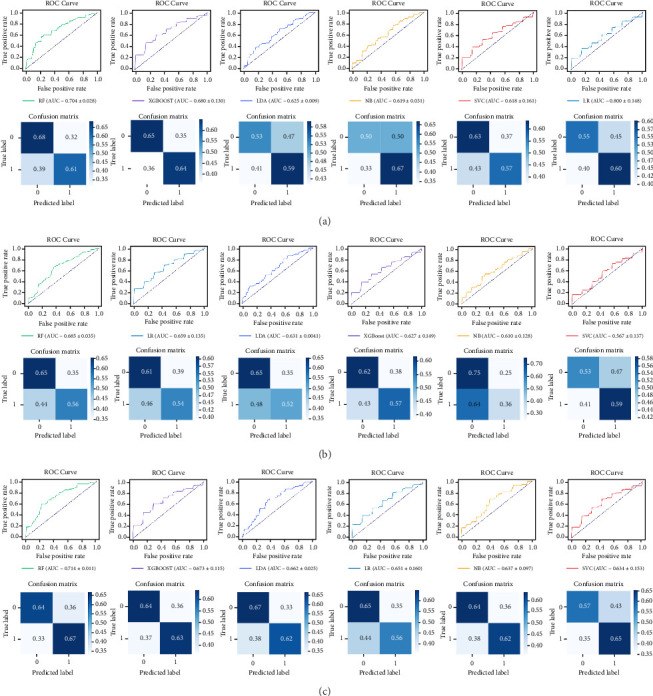
The performance of six machine learning algorithms. (A) Eye tracking features with significant group differences were chosen as input. (B) VEP features were used as input. (C) Combined eye tracking and VEP features were utilized as model input. Above is the ROC curve, and below is the confusion matrix. AUC, area under the curve; LDA, linear discriminant analysis; LR, logistic regression; NB, naive Bayes; RF, random forest; ROC, receiver operating characteristic; SVC, support vector classification; VEP, visual evoked potential; XGBoost, extreme gradient boosting.

**Figure 5:**
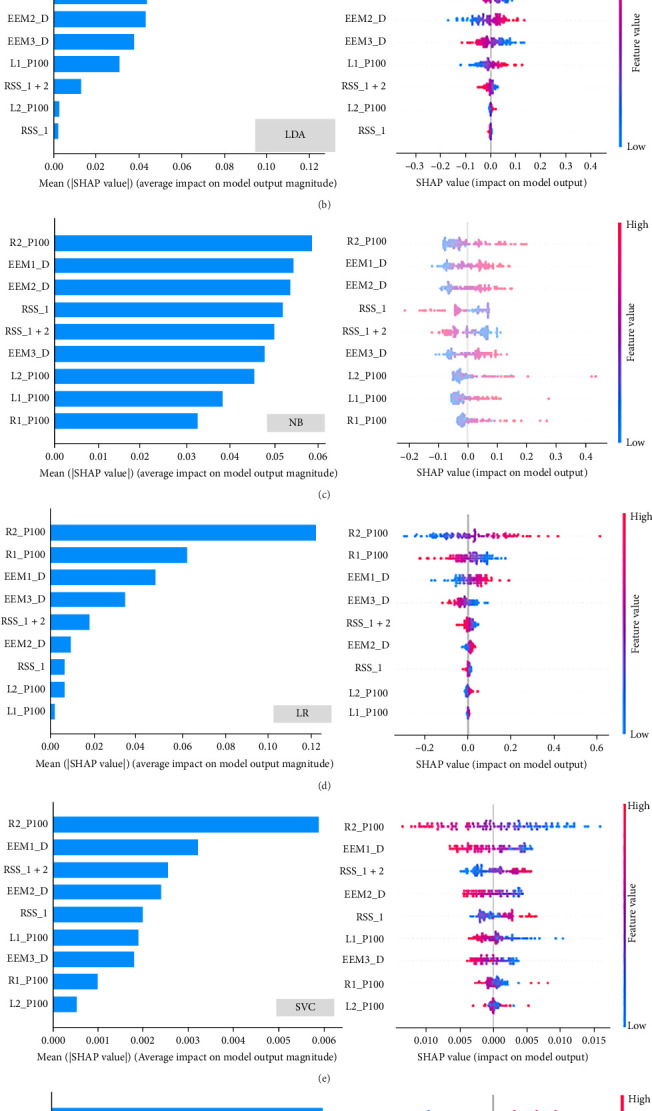
Feature importance is determined by Shapley Additive ExPlanations (SHAP) values. The top eye tracking and visual evoked potential features identified by SHAP for the classification model are ordered from most to least important. (A) RF model. (B) LDA model. (C) NB. (D) LR model. (E) SVC model. (F) XGBoost model. On the left, feature importance is determined by calculating the mean of absolute SHAP values for each feature. Larger bars indicate the feature's importance in discriminating between depression and nondepression. The right side shows the summary plot of the contributions of all nine features in the combined model. Each point corresponds to one student. Red and blue correspond to higher and lower values, respectively. EEM, eye movement; L1P100, left eye 1, P100; L2P100, left eye 2, P100; LDA, linear discriminant analysis; LR, logistic regression; NB, naive Bayes; R1P100, right eye 1, P100; R2P100, right eye 2, P100; RF, random forest; RSS, responsiveness of search scores; SHAP, SHapley Additive exPlanations; SVC, support vector classification; VEP, visual evoked potential; XGBoost, extreme gradient boosting.

A total of five eye tracking features and four VEP features exhibiting significant alterations were selected as inputs for the machine learning algorithms. Prior to model training, *Z*-score normalization was applied to mitigate biases among the features. During the training and testing phases of the classification model, we initially assessed the features extracted from the eye tracking data. The RF model exhibited superior classification performance (AUC = 0.704, accuracy = 0.645, precision = 0.639, recall = 0.606, and F1 = 0.620), compared to LDA, NB, LR, XGBoost, and SVM. Initially, we evaluated features derived from VEPs, where the RF model achieved the highest accuracy of 0.609 and an AUC of 0.665. Subsequently, six classification models were developed by integrating eye tracking and VEP features. Consistently, the RF classifier (AUC = 0.714, accuracy = 0.652, precision = 0.630, recall = 0.667, and F1 = 0.646) outperformed the other five classifiers. Overall, the RF model demonstrated superior classification performance relative to the other five machine learning algorithms (Table 3 and Figure 4). Models utilizing combined features showed enhanced performance (AUC = 0.714, accuracy = 0.652, precision = 0.630, recall = 0.667, and F1 = 0.646) compared to those based solely on eye tracking or VEP features. Utilizing the SHAP method, we identified that the P100 latency in the right eye (R2P100) was the most significant feature across all machine learning models (Figure 5).

## 4. Discussion

To the best of our knowledge, this is the first study to integrate eye tracking, VEP, and auditory P300 features with six distinct machine learning algorithms to differentiate between individuals with and without depression among Chinese university students. This study yielded three primary findings. First, medical students with depression exhibited lower RSS values, higher D values, and prolonged P100 latencies, indicating alterations in attentional engagement and cognitive processing associated with depression. Second, the *D* values and P100 latency in the right eye were significantly associated with the severity of depression, further supporting attention and visual information processing impairment in individuals with depression. Third, the integration of eye tracking and VEP features demonstrated superior performance in depression detection, achieving a higher AUC of 0.714. SHAP analysis identified P100 latency in the right eye as the most important feature in the combined model, implicating reduced right-lateralized responsivity in the visual cortex and right-hemisphere hypoactivation. Consequently, these findings support the integration of multiple neurophysiological measurements with six distinct machine learning algorithms as an effective strategy for identifying depression among medical students in China.

The present study found lower RSS scores and elevated *D* values in medical students with depression, suggesting reduced concentration, deficits in cognitive processing speed, and working memory. These results align with the findings of several prior studies. Individuals with depression typically exhibit eye tracking patterns characterized by shorter fixation durations on positive stimuli, reduced scan path lengths, and fewer fixations [25–27]. Research on exploratory eye tracking tasks has shown that discriminant analysis *D* values are consistently higher in the depression group compared to controls in repeated assessments [28]. Furthermore, a meta-analytic review has indicated that depression is linked to heightened attention towards dysphoric stimuli and decreased attention toward positive stimuli [29]. University students with depression show eye tracking patterns characterized by negative emotions and changed scanning speeds [9, 30]. Our results support these findings and emphasize attentional biases as a key aspect of depression. Additionally, this study did not observe significant differences in the MESL during eye tracking tests between the depression and control groups, differing from some earlier studies [28]. This inconsistency may be attributed to variations in the task's requirements or differences in the eye tracking equipment. Therefore, further exploration and validation in a larger sample are needed in the future.

An intriguing finding from the current study is the prolonged P100 latencies in both eyes (R1P100, R2P100, L1P100, and L2P100) for medical students with depression, which may indicate slowed information processing, impaired visual attention, and hypoarousal. Previous studies have demonstrated that individuals with depression are susceptible to distraction during visual stimulus tasks and exhibit extended P100 latencies [10, 31, 32]. This delay in information processing has the potential to hinder the acquisition of new knowledge, particularly in an academic setting. Furthermore, the VEP recorded from the visual cortex in individuals with depression exhibited significantly reduced amplitudes compared to controls. This reduction may be attributed to alterations in “cognitive neuronal pools” or disruptions in internal homeostasis [33]. Bruder and Przegaliński et al. have demonstrated the prolonged P100 latencies, which are associated with reduced serotonergic and dopaminergic activity [33, 34]. The LR analysis revealed that the P100 latencies were associated with depression severity, providing neurophysiological evidence of impaired attention and visual information processing. In addition, the latency of VEPs may be associated with the degree of arousal. Consequently, we speculate that the medical students experiencing depression may exhibit hypoarousal. Our study contributes to the understanding of impairments in higher cognitive processes in depression, especially in response to visual stimuli. From the perspective of information processing, prolonged P100 latency may be associated with the reduced activity of the visual cortex, suggesting impaired early visual processing.

The present study showed no significant difference in auditory P300 between medical students with depression and controls. Auditory P300 are endogenous potentials that serve as indicators of the brain's cognitive functions [35]. This component has been extensively investigated in the context of depression, providing insights into the neural processes underlying depression [12]. Prior research has identified the N200 and P300 components associated with cognitive flexibility [36]. The studies have demonstrated that the amplitude of the error-related negativity associated with error processing is reduced in depression [37]. This indicates that altered neural responses to errors or negative feedback in depression. Furthermore, evidence reveals that these depressed medical students experience prolonged P3 latencies, especially when responding to positive emotional stimuli, suggesting a delayed cognitive processing of mood-incongruent information [38, 39]. Several factors may explain the absence of group-level differences observed in our study. First, participants with a PHQ-9 score of ≥ 5 were categorized as having depression in the study. Ghazisaeedi et al. [40] have demonstrated that the PHQ-9 is effective for screening depression among medical university students when employing a cut-off score of ≥ 5. However, a PHQ-9 score of ≥ 5 may not necessarily indicate clinically significant depression, which could account for the lack of statistically significant changes in auditory P300 characteristics. This observation is consistent with previous research suggesting that variations in P300 amplitude are more closely associated with the severity of depressive symptoms rather than cognitive status itself [41]. Second, the auditory oddball paradigm utilized in this study, despite its widespread use, may have limited sensitivity in detecting subtle cognitive processing deficits, particularly in subclinical populations [42, 43]. Recent studies have indicated that a modified bimodal oddball task, which combines auditory and visual stimuli, may provide greater sensitivity in identifying cognitive alterations linked to mild depression [44]. Additional research has investigated the application of three-tone oddball paradigms, indicating that substituting traditional auditory stimuli with novel sounds may offer further benefits [45]. Finally, technical variables such as trial count, task duration, or electrode configuration might have diminished the signal-to-noise ratio, thus reducing the detectability of subtle group effects. Further investigation is necessary to elucidate these factors contributing to nonsignificant findings.

Notably, our study reveals that the RF classifiers utilizing combined eye tracking and VEP feature sets outperformed those using a single-feature set, achieving an AUC of 0.714. As an ensemble machine learning algorithm based on decision trees, RF can effectively address nonlinear effects and complex interactions among variables. It possesses the capability to mitigate overfitting and enhance model accuracy [46, 47]. RF demonstrates significant advantages over alternative algorithms, notably its cost-effectiveness and interpretability [47]. Previous research has demonstrated that RF achieved notable performance, such as 92% sensitivity and 88% specificity in diagnosing nonsevere depression [48], as well as up to 95% accuracy in predicting depression among children and adolescents [49]. These findings underscore the superior efficacy of RF relative to other classifiers, highlighting its potential for developing precise and reliable depression detection models. Consequently, RF emerges as an efficient and valuable tool for identifying critical features and optimizing classification accuracy in depression. Moreover, the RF model that integrates eye tracking and VEP features demonstrated superior performance compared to models utilizing a single feature set. These results underscore the enhanced predictive capability of the integrated model. A possible explanation for this improvement is that the incorporation of multiple neurophysiological measures offers a more comprehensive assessment of depression, thereby enhancing the accuracy and reliability of predictions. Although previous studies have demonstrated that the PHQ-9 is suitable for screening depressive symptoms in nonclinical populations [50], it is also important to note that depressive status in this study was determined solely using the PHQ-9, which serves as a screening rather than a diagnostic instrument. Thus, the observed associations should be interpreted as reflecting preliminary depressive symptoms rather than clinically confirmed diagnoses. Overall, the integration of six distinct machine learning methodologies alongside multiple neurophysiological measures in this study contributes to the advancement of more effective classification frameworks.

Interestingly, the SHAP analysis identified the P100 latency in the right eye as potentially the most significant feature in the combined model for predicting depression. SHAP, rooted in cooperative game theory, assigns Shapley values to each feature. This precisely quantifies each feature's contribution and provides a transparent measure of importance. This approach provides a clear and interpretable understanding of how each feature influences the outcome, distinguishing it from simpler importance metrics. The P100 component of the VEP reflects the processing speed of the primary visual cortex. It is noteworthy that stimuli presented in the left visual field are predominantly processed by the right visual cortex. Consequently, when stimuli are introduced to the left visual field and recorded via the right-eye pathway, the P100 latency can serve as an indirect indicator of the processing efficiency of the right hemisphere [10]. The SHAP analysis consistently identified the P100 latency in the right eye as potentially the most significant feature in six combined models for predicting depression. A plausible explanation for our findings is provided by the right hemisphere hypothesis of depression [51], which suggests that the right cerebral hemisphere plays a central role in the processing of negative emotions, attentional vigilance, and the withdrawal motivational system. Depression is often associated with a hyperactive withdrawal system and a persistent negative emotional bias, which may impose a chronic processing burden on the right hemisphere [52]. Consequently, when faced with visual input, individuals with depression may experience diminished processing efficiency in the already dysregulated right hemisphere. In our study, this inefficiency manifested as prolonged P100 latency, supporting the notion that altered early-stage visual processing may reflect broader hemispheric dysfunction. Therefore, the significance of right-eye P100 latency during left visual field stimulation may not solely indicate perceptual asymmetry but also serve as a potential neurophysiological marker of core right-hemispheric impairment associated with depression [33]. Although alternative explanations, such as impaired interhemispheric transfer, cannot be completely dismissed, the specificity of this feature aligns strongly with theories that emphasize the critical role of the right hemisphere in depression. This finding provides valuable insights into the neurophysiological underpinnings of depression, particularly when analyzed through the framework of cognitive neuroscience theories on hemispheric specialization.

## 5. Limitations

Several limitations should be addressed. First, the sample size was relatively small; future studies should include larger samples to produce more precise effect-size estimates. The cross-sectional design limits our ability to conclude the temporal dynamics of depression-related changes in eye tracking and VEP features. Expanding the sample size and utilizing longitudinal studies could strengthen the robustness of the results. Second, the current model outcomes have not been validated with external datasets. Future research should prioritize the validation of these findings across diverse datasets to ensure the reliability and generalizability of the models. Third, the PHQ-9 serves as a screening tool and does not replace the gold standard of clinical diagnosis. Future studies should incorporate clinical diagnostic interviews, such as the Hamiltondepression rating scale, to more robustly validate depressive status. Fourth, although six machine learning models exhibited moderate accuracy, there is considerable room for improvement. Future research should explore deep learning techniques to further enhance the predictive power.

## 6. Conclusions

Chinese medical students experiencing depression showed lower RSS scores, higher D values, and prolonged P100 latencies in both eyes, suggesting altered higher cognitive processes in response to visual stimuli, as well as impairments in attention and visual information processing associated with depression. RF model incorporating both eye tracking and VEP features outperformed models that used a single-feature set. These findings underscore the potential benefits of employing multiple neurophysiological features within a multimodal framework as an effective strategy for the early detection of depression among Chinese medical students. Furthermore, the identification of the P100 latency in the right eye as the most significant feature offers insights into the neurophysiological mechanisms underlying depression.

## Figures and Tables

**Table 1:** Demographic, eye tracking, and visual evoked potential characteristics of controls and depression among Chinese medical students.

Characteristic	Control (*N* = 72)	Depression (*N* = 66)	t/*χ*^2^	*p*-Values
Demographic characteristics				
Gender (female/male)	56/16	50/16	0.079	0.841
Age (year)	20.89 ± 1.87	20.45 ± 1.76	1.405	0.162
Education (years)	15.15 ± 1.87	14.79 ± 1.58	1.230	0.221
PHQ-9 score	1.65 ± 1.33	8.53 ± 2.99	−17.168	<0.001
GAD-7 score	0.75 ± 1.14	5.83 ± 3.46	−11.380	<0.001
ISI score	2.63 ± 1.95	7.83 ± 4.47	−8.731	<0.001
Eye tracking				
RSS1	2.6 ± 1.1	2.2 ± 0.77	2.499	0.014⁣^*∗*^
RSS (1 + 2)	5.33 ± 1.65	4.68 ± 1.5	2.417	0.017⁣^*∗*^
EEM1 D	3.87 ± 1.42	4.47 ± 1.32	−2.579	0.011⁣^*∗*^
EEM2 D	3.84 ± 1.43	4.45 ± 1.4	−2.516	0.013⁣^*∗*^
EEM3 D	3.83 ± 1.5	4.38 ± 1.41	−2.219	0.028⁣^*∗*^
Visual evoked potentials				
Right eye 1, P100	110.21 ± 6.52	112.84 ± 8.35	−2.071	0.04⁣^*∗*^
Right eye 2, P100	109.77 ± 6	114.11 ± 8.08	−3.607	<0.001⁣^*∗∗∗*^
Left eye 1, P100	111.64 ± 7.11	114.74 ± 7.63	−2.473	0.015⁣^*∗*^
Left eye 2, P100	112.08 ± 6.43	114.86 ± 8.81	−2.137	0.034⁣^*∗*^

*Note:* Continuous data are presented as mean (SD) and categorical data as *n* (%).

Abbreviations: EEM, eye movement; GAD-7, generalized anxiety disorder-7; ISI, insomnia severity index; PHQ-9, patient health questionnaire-9; RSS, responsiveness of search scores; VEP, visual evoked potential.

*⁣* ^*∗*^ *p* < 0.05.

*⁣* ^*∗∗*^ *p* < 0.01.

*⁣* ^*∗∗∗*^ *p* < 0.001.

**Table 2:** Multivariate logistic regression analysis.

Variable	*β*	SE	Wald	OR (95% CI)	*p*-Value
EEM1 D	0.278	0.141	3.867	1.320 (1.001–1.741)	0.049
Right eye 1, P100	−0.117	0.055	4.504	0.890 (0.798–0.991)	0.034
Right eye 2, P100	0.197	0.06	10.89	1.217 (1.083–1.368)	0.001

Abbreviation: EEM, eye movement.

**Table 3:** Performances of six machine learning algorithms based on eye tracking, visual evoked potential, and combined features.

Feature set	Modal	AUC	Accuracy	Precision	Recall	F1-score
Eye tracking	RF	0.704	0.645	0.639	0.606	0.620
	XGBoost	0.680	0.641	0.658	0.639	0.632
	LDA	0.625	0.588	0.537	0.591	0.557
	NB	0.619	0.579	0.549	0.666	0.598
	SVC	0.618	0.603	0.604	0.571	0.577
	LR	0.600	0.577	0.587	0.603	0.584

VEPs	RF	0.665	0.609	0.596	0.561	0.577
	LR	0.659	0.574	0.593	0.538	0.552
	LDA	0.631	0.587	0.594	0.515	0.537
	XGBoost	0.627	0.594	0.605	0.571	0.579
	NB	0.610	0.565	0.567	0.364	0.435
	SVC	0.567	0.557	0.566	0.588	0.563

Eye tracking and VEPs	RF	0.714	0.652	0.630	0.667	0.646
	XGBoost	0.673	0.636	0.651	0.632	0.631
	LDA	0.662	0.645	0.628	0.621	0.622
	LR	0.651	0.603	0.611	0.560	0.568
	NB	0.637	0.632	0.616	0.621	0.617
	SVC	0.634	0.613	0.612	0.651	0.624

Abbreviations: AUC, area under the curve; LDA, linear discriminant analysis; LR, logistic regression; NB, naive Bayes; RF, random forest; SVC, support vector classification; VEP, visual evoked potential; XGBoost, eXtreme gradient boosting.

## Data Availability

The data that support the findings of this study are available on request from the corresponding author. The data are not publicly available due to privacy or ethical restrictions.
